# Biomarkers in Irritable Bowel Syndrome: Bridging Gut–Brain Mechanisms to Precision Care

**DOI:** 10.1007/s11894-026-01053-2

**Published:** 2026-08-15

**Authors:** Manjeet Kumar Goyal, Omesh Goyal, Rishi Chowdhary, Tanisha Sehgal, Gayatri Brahmandam, Anushri Parikh, Insiya Rampurawala, Ashita Rukmini Vuthaluru, Prerna Goyal, Abdulfattah Issak

**Affiliations:** 1 Department of Internal Medicine, Cleveland Clinic Akron General Hospital, 1 Akron General Avenue, Akron, OH 44307 USA; 2 Department of Gastroenterology, Dayanand Medical College and Hospital, Ludhiana, Punjab 141 001 India; 3 Department of Medicine, MetroHealth Medical Center, Cleveland, OH 44109 USA; 4 Department of Medicine, Dayanand Medical College and Hospital, Ludhiana, 141 001 India; 5 Department of Medicine, Adichunchanagiri Institute of Medical Sciences, Mandya, Karnataka 571448 India; 6 Department of Medicine, Medical College Baroda, Vadodara, Gujarat 390001 India; 7 Department of Internal Medicine, J.S.S. Medical College, Mysuru, Karnataka 570015 India; 8 Department of Anaesthesiology and Critical Care, All India Institute of Medical Sciences, New Delhi, 110029 Delhi India; 9 Department of Medicine, R.G. Stone and Super Specialty Hospital, Ludhiana, 141 002 India

**Keywords:** DGBI, Biomarkers, Anti-CdtB antibodies, Lactobacillus, Multi-omics

## Abstract

**Purpose of Review:**

Irritable bowel syndrome is a common disorder of gut-brain interaction characterized by chronic abdominal pain and altered bowel habits in the absence of structural pathology. Its pathophysiology reflects a complex interplay between the central nervous system, enteric nervous system, microbiota, immune signaling, epithelial barrier dysfunction etc. Despite advances in the understanding of the mechanisms causing IBS, the diagnosis of IBS remains primarily symptom-based, necessitating the use of objective tools to improve diagnostic precision and personalized management.

This review synthesizes highlights existing literature on IBS biomarkers, including inflammatory markers (cytokines, C-reactive protein), microbial signatures (gut microbiota composition, anti-CdtB and anti-vinculin antibodies), metabolic indicators (bile acids, short-chain fatty acids), and markers of intestinal permeability (zonulin) to assess their applications in the diagnosis and management of IBS. We also examined emerging multi-omics technologies that identify the complex patterns linking the gut, the brain and the microbiota. Early research focusing on single biomarkers demonstrated limited diagnostic utility due to the heterogeneous and multifactorial nature of IBS. Recent advances support the development of composite, mechanism-based biomarker panels that integrate multiple biological domains, offering improved representation of underlying pathophysiological processes.

**Summary:**

These developments mark a shift from a single-marker approach toward multidimensional biomarker strategies. This narrative review synthesizes current evidence and proposes a framework for integrating biomarkers into clinical practice. Biomarkers are best utilized following a positive symptom-based diagnosis and exclusion of alarm features, to stratify patients into biologically relevant subtypes and guide targeted therapy. This supports a transition from exclusion-based diagnosis of IBS towards a positive and more precise one.

**Supplementary Information:**

The online version contains supplementary material available at 10.1007/s11894-026-01053-2.

## Introduction

Functional gastrointestinal (GI) disorders, now known as ‘disorders of gut-brain interaction’ (DGBI), represent a group of chronic GI disorders with multiple pathophysiological mechanisms including altered GI motility, visceral hypersensitivity, mucosal and immune function, gut microbiota, central nervous system (CNS) processing, etc. [1]. These disorders are no longer viewed as isolated gut pathologies but rather as manifestations of dynamic, bidirectional gut–brain axis dysfunction.

Amongst DGBIs, Irritable Bowel Syndrome (IBS) is the most prevalent, affecting between 10 and 15% of the general population [1]. Despite this high prevalence, it has not been easy to diagnose IBS accurately. This is partly due to the nonspecific and fluctuating nature of the symptoms, and its overlap with other DGBIs which defy a neat classification [2]. Historically described as “mucus colitis” or “spastic colon” in 19th and 20th-century reports, IBS was long regarded as a diagnosis of exclusion rather than a positively defined entity [3].

Few decades ago, Manning and his colleagues identified symptom clusters like pain related to defecation, altered stool frequency and form, abdominal distension, and a sense of incomplete evacuation, which were more reliable than testing to exclude other organic causes [3]. This facilitated a symptom-based approach to diagnosing IBS. Subsequent iterations of the ROME criteria and the DGBI framework have modeled IBS as a disorder shaped by biological, psychological, and social factors [4] as shown in Supplementary Fig. 1.

The diagnosis of IBS still relies on symptom-based criteria, normal basic investigations, and exclusion of alarm features [3]. Symptom criteria alone have limited ability to differentiate IBS from overlapping conditions, such as functional dyspepsia (FD), small intestinal bacterial overgrowth, subtle IBD, celiac disease, non-celiac gluten sensitivity (NCGS), etc. [5]. This diagnostic uncertainty frequently results in delayed diagnosis, repeated testing, and suboptimal symptom control, underscoring the need for more objective tools.

Early biomarker research was largely unsuccessful, in part because it sought a single diagnostic marker for a disorder driven by multiple interacting pathways. This approach proved inadequate for a biologically heterogeneous syndrome such as IBS, where different patients may exhibit distinct dominant pathways including dysbiosis, bile acid dysregulation, immune activation, visceral hypersensitivity etc.

Accordingly, more recent efforts have shifted toward identifying multidimensional biomarker panels that reflect underlying biological heterogeneity. This review summarizes the limitations of current symptom-based diagnostic strategies in IBS and evaluates the evolving role of a biomarker-driven approach.

## Materials and Methods

This narrative review was informed by a targeted, non-systematic literature search of PubMed/MEDLINE and Scopus from database inception to [insert final search date]. Search terms included combinations of “irritable bowel syndrome,” “IBS,” “biomarkers,” “microbiome,” “gut–brain axis,” “multi-omics,” and “artificial intelligence.” Additional relevant publications were identified through manual screening of the reference lists of key articles. Original studies evaluating diagnostic, prognostic, predictive, or mechanistic biomarkers were considered alongside relevant validation studies, systematic reviews, meta-analyses, clinical practice guidelines, consensus statements, and landmark publications. Evidence was selected according to its clinical relevance, methodological contribution, and importance to the evolving biomarker framework and was synthesized narratively by biological domain. The review was not conducted according to a prespecified systematic-review protocol; study screening was not performed in duplicate, and formal risk-of-bias and certainty-of-evidence assessments were not undertaken. Accordingly, this article should be interpreted as a narrative synthesis rather than a systematic or exhaustive review of the literature.

### Conceptual Framework of IBS as a Disorder of Gut-Brain Interaction

Over the last 50 years, IBS has shifted from being viewed as a disorder without structural changes to one with subtle gut-brain axis dysfunction. This conceptual shift was first formalized in the Rome IV criteria, which redefined functional GI disorders as “disorders of gut–brain interaction” [6]. The recently released Rome V criteria build on this foundation, moving beyond categorical, symptom-threshold-based definitions toward a multidimensional and clinically operational framework [7]. For IBS specifically, Rome V reintroduces abdominal pain, lowers the symptom frequency threshold to at least 3 days per month. It introduces the requirement that symptoms be intermittent rather than continuous, a change that fundamentally redefines IBS as a time-structured disorder and distinguishes it from centrally mediated abdominal pain disorders, where pain is continuous or near-continuous [7]. These recalibrations restore diagnostic sensitivity that was lost under the stricter Rome IV thresholds while improving mechanistic specificity, and they carry direct implications for how biomarker-based subtyping strategies, as discussed in this review, should be aligned with the updated diagnostic boundaries of IBS. The ROME V criteria have fully transformed functional gastrointestinal disorders into disorders of gut brain interaction [6, 7]. 

These conditions arise from bidirectional signaling between the central and enteric nervous systems, influenced by factors such as the microbiota, the mucosal immune system etc. [8, 9]. Disruptions in this network lead to abnormalities in visceral sensation, motility, secretion, and pain processing, supported by converging evidence from neuroimaging, neurogastroenterology, and microbiome research. Food allergies and food triggers may alter symptom expression in IBS patients, further complicating the diagnosis [10]. Supplementary Fig. 2 outlines the mechanisms that may influence the pathophysiology of IBS.

Importantly, therapeutic response in IBS is highly variable. Although therapies targeting bowel habits may benefit some patients, many patients show partial or no improvement, pointing to underlying biological heterogeneity rather than treatment failure [11, 12]. This therapeutic variability necessitates the need for biomarkers that represent dominant pathophysiological drivers in individual patients.

Human studies have demonstrated reproducible alterations in central pain-processing networks and stress-responsive brain regions in IBS, alongside gut permeability, immune activation, and patient presentations [13, 14]. These findings support multidimensional biomarker approaches rather than reliance on single markers.

Advances in gut-focused research further implicate the microbiome in host metabolism, gut motility, permeability, and visceral sensitivity [15]. Mechanisms include 5-HT receptor signaling, activation of the hypothalamic-pituitary axis, immune dysregulation, and epithelial barrier dysfunction (tight junction protein expression), as well as microbial metabolites (short-chain fatty acids, secondary bile acids, tryptophan metabolites) [16].

Despite strong biological plausibility, clinically validated biomarkers for IBS have remained elusive due to several factors. This is largely due to the dynamic and context-dependent nature of gut–brain interactions, influenced by stress, diet, microbiota composition, and psychosocial factors. Consequently, single-point measurements often lack sensitivity and reproducibility. A biologically informed framework that defines IBS endotypes based on dominant mechanisms offers a more promising path toward precision diagnostics and targeted therapy.

### Rationale for Biomarker Development in IBS

The development of biomarkers for IBS stems from the limitations of symptom-based criteria, particularly the suboptimal specificity and substantial symptom overlap with other DGBIs [17]. In clinical practice, the exclusion-based diagnosis of IBS necessitates numerous tests, which can often be expensive and pose an additional burden [17]. Thus, utilizing the newer diagnostic tools, such as biomarkers in the diagnosis of IBS can help eliminate excessive testing. Biomarker-based approaches offer a more targeted alternative by reflecting underlying pathological mechanisms, improving diagnostic accuracy, and enabling identification of IBS subtypes to guide personalized therapies [18] These serum biomarkers have also shown value in randomized controlled trials investigating new interventions for IBS. Supplementary Table 1 summarizes the various categories of biomarkers and their classification.

Additionally, IBS clinical trials are hindered by high placebo response rates, which are at around 40%, complicating the interpretation of treatment efficacy [19]. Emerging evidence suggests that specific biomarker profiles may help identify placebo responders and refine trial design, thereby enhancing signal detection [19]. Importantly, biomarkers may also correlate with disease severity and patient-reported outcomes, providing objective endpoints to complement subjective symptom measures. However, their integration into clinical practice requires robust validation and demonstration of clinical utility [12, 19].

### Types of Biomarkers

A Inflammatory Biomarkers

Emerging evidence discusses the role of low-grade inflammation in the pathogenesis of IBS, specifically epithelial dysfunction and visceral hypersensitivity [20]. Approximately 70% of patients with IBS demonstrated low-grade inflammation along their intestinal mucosa and lamina propria, resulting in immune activation and release of pro-inflammatory cytokines, which have the potential to serve as biomarkers [20].

Key cytokines, including TNFα, IL-1β, IL-6, and IL-10, are elevated in IBS-D and post-infectious IBS (PI-IBS), but not in IBS-C [21, 22]. Gender based differences have also been demonstrated in IBS, where women with IBS-D demonstrated higher TNFα and lower IL-10 levels as compared to other subtypes [21]. Studies have also reported elevated levels of IL-5 and IL-9, which are typically secreted by mast cells, which are indicated to be important mediators in the pathophysiology of IBS [23].

Some inflammatory pathways overlap with IBD, resulting in increased TH1, TH2, and TH17 cytokines also elevated in IBS [24]. Another frequently studied inflammatory biomarker, fecal calprotectin, is found in neutrophils, monocytes, and macrophages. It has a sensitivity and specificity of around 95% and is used to differentiate IBS from IBD [18]. Faecal calprotectin is primarily useful for identifying clinically significant intestinal inflammation rather than confirming IBS. A low concentration commonly below 50 µg/g makes active inflammatory bowel disease unlikely, whereas an elevated result warrants further evaluation for organic disease but does not independently exclude IBS [25].

Inflammatory biomarkers may aid in diagnosis, subtype differentiation, and assessment of symptom severity, with correlations observed between cytokine levels, mast cell activity, and clinical features such as bloating [21]. Serum inflammatory biomarker levels have also been hypothesized to be correlated with quality of life in IBS patients; however, the available evidence has shown conflicting findings [21].

The most comprehensive synthesis to date was provided by Burns et al., who included 124 studies encompassing 14,930 patients with IBS. Compared with healthy controls, patients with IBS had higher circulating concentrations of tumour necrosis factor-α, interleukin-6, and interferon-γ, as well as higher faecal calprotectin and lower faecal valerate concentrations. Faecal calprotectin concentrations remained significantly lower in IBS than in organic gastrointestinal diseases, supporting its greater utility for identifying intestinal inflammation than for confirming IBS. Subgroup analyses also identified higher interleukin-6 and lower serum albumin concentrations in IBS-D than in healthy controls. However, moderate-to-high heterogeneity, differences in assay methodology, and the absence of validated diagnostic thresholds limit the immediate clinical applicability of these findings. Collectively, the results support the presence of measurable biological signatures in IBS but do not establish any individual marker as a standalone diagnostic test [22].

However, their clinical application is currently limited by the absence of standardized cutoff values and the multifactorial nature of IBS. The integration of biomarkers into clinical practice requires robust validation and demonstration of clinical utility [20, 24].

B Microbial and Post-Infectious Biomarkers

Alterations in gut microbiota further contribute to IBS pathogenesis through effects on immune activation, bile metabolism, and bile acid production, resulting in increased severity of IBS symptoms [26].

Studies have noted substantial differences between the diversity of microbiota found in the mucosal and fecal samples in IBS patients. Changes in dominant phyla such as Firmicutes and Bacteroidetes have been reported, with reduced levels of beneficial genera like *Bifidobacterium* and *Lactobacillus* across IBS subtypes while *Actinobacteria* and *Proteobacteria* levels were unchanged [16]. This was associated with inflammation and symptom severity [26].

Additionally, the gut microbial diversity can be altered following an acute gastrointestinal infection. The fecal microbiota composition in PI-IBS is significantly different from the microbiota of healthy individuals [26]. The most commonly implicated microorganism in PI-IBS is *Campylobacter jejuni* [26]. However, findings have been inconsistent, with some studies suggesting overlap with other IBS subtypes, while others report reduced microbial diversity and specific shifts in bacterial populations. A study identified 27 genus-like groups contributing to an Index of Microbial Dysbiosis in PI IBS, characterized by elevated levels of Bacteroidetes and Prevotella species, which was similar to IBS D [16]. Another study found that the microbial diversity in PI-IBS differed from other subtypes of IBS. Reduced levels of clostridial species were reported compared to healthy controls [27].

There are several serum antibody biomarkers that can help diagnose PI-IBS and IBS-D. Elevated antibodies against cytolethal distending toxin (CdtB), produced by bacterial organisms, including *Campylobacter*,* Salmonella*,* E coli*, and *Shigella*, demonstrate high specificity of 90% but a modest sensitivity of 43% [18, 28]. It was also hypothesized that exposure to CdtB resulted in development of antibodies that attacked vinculin proteins found on intestinal epithelial cells. This resulted in loosened adherence across these epithelial cells, which are responsible for symptoms seen in IBS-D patients [28]. These anti-vinculin antibodies are also found elevated in IBS-D patients and have a specificity and sensitivity of around 80% and 30% [18]. These biomarkers had overlapping values seen in other conditions such as celiac disease highlighting ongoing challenges in clinical translation [28].

Lactulose breath testing (LBT) has limited diagnostic specificity but may have some utility in assessing methane production in IBS-C, though evidence regarding its clinical relevance remains inconsistent [17]. However, even these biomarkers demonstrate only modest sensitivity, and their diagnostic performance varies across populations due to inter-individual variability influenced by diet, geography, and sampling methods [29].

C Metabolic and Bile Acid–Related Biomarkers

Alterations in bile acid (BA) metabolism represent another important pathway in IBS pathophysiology. Bile acids regulate lipid absorption, gastrointestinal motility, and secretion of water, mucus, and electrolytes [30].

Patients with IBS-D typically exhibit increased total and primary fecal bile acids with relative reductions in secondary bile acids, although findings regarding specific subtypes remain inconsistent [31]. Bile acid malabsorption is observed in approximately 30% of IBS-D cases and is associated with increased hepatic bile acid synthesis, reflected by elevated serum levels of 7α-hydroxy-4-cholesten-3-one (7αC4) [30]. Conversely, fibroblast growth factor 19 (FGF19), which suppresses bile acid synthesis, is reduced in IBS-D [30].

In contrast, IBS-C has been associated with reduced fecal bile acids, decreased C4, and elevated FGF19 levels, which is justified given the reduced colonic motility [32]. These findings highlight the distinct underlying drivers in symptom production across IBS subtypes.

Short-chain fatty acids (SCFAs), including acetate, propionate, and butyrate, are additional microbial metabolites implicated in IBS [18]. Altered SCFA levels have been reported across subtypes, with evidence suggesting subtype-specific variations, although the exact findings remain elusive [33]. Potential mechanisms of SCFAs include modulating intestinal inflammation, gut integrity, and gut motility [33]. A meta-analysis reported elevated levels of propionate in all subtypes of IBS. On further subgroup analysis, propionate and butyrate were significantly lower in IBS-C, and butyrate was elevated in IBS-D [34].

The metabolic markers mentioned in this section can diagnose patients with IBS and also help develop personalized treatment for IBS-C and IBS-D patients [30, 32]. Table 1 lists the inflammatory, microbial, and metabolic biomarkers that have been understood thus far.

**Table 1:** Key inflammatory, microbial, and metabolic biomarkers studied in IBS

Category	Biomarker	Sample Type	IBS-C Findings	IBS-D Findings
Inflammatory	TNF-α, IL-1β, cortisol, IL-10	Serum	Elevated → low-grade inflammation [53]	Elevated, low-grade inflammation
	IL-5, IL-9	Serum/Mast cells	Positive correlation with ↑ bloating frequency	Positive correlation with ↑ bloating frequency
	CRP	Serum	Elevated → suggests microinflammation	Elevated, suggests microinflammation
	ESR	Serum	Elevated → suggests microinflammation	Elevated, suggests microinflammation
	Fecal calprotectin	Stool	Sensitivity/specificity ~ 85% (used to exclude IBD) [25]	Same utility (exclude IBD)
Microbial	Gut microbiota composition	Stool/mucosal biopsy	↓ Bifidobacterium, ↓ Lactobacillus	↓ Firmicutes, Fusobacteria; ↑ Bacteroides; no significant change in Actinobacteria & Proteobacteria
	Anti-CdtB antibodies	Stool	-	Specificity ~ 90%, Sensitivity ~ 43% [54]
	Anti-vinculin antibodies	Stool	-	Specificity ~ 80%, Sensitivity ~ 30%
	Lactulose breath test	Breath (methane & hydrogen)	↑ Methane directly correlates with severity of IBS-C	↑ Hydrogen (implied association with IBS-D) [55]
Metabolic	Primary & secondary bile acids	Stool	↑ Secondary bile acid (LCA), ↓ DCA, ↓ total bile acids vs. controls	↑ Primary bile acids vs. controls [31]
	C3, C4	Serum	↓ C4 → marker of decreased bile acid production	↑ C4 → marker of increased bile acid production
	FGF19	Serum	↓ FGF19 vs. controls → reduced negative feedback to C4	↑ FGF19 vs. controls (feedback response)
	Short-chain fatty acids (SCFAs: acetate, propionate, butyrate)	Stool	↓ Propionate, ↓ Butyrate	↑ Butyrate

*CRP* C-reactive protein, *CdtB* cytolethal distending toxin B, *DCA* deoxycholic acid, *ESR* erythrocyte sedimentation rate, *FGF19* fibroblast growth factor 19, *IL* interleukin, *IBD* inflammatory bowel disease, *IBS-C* constipation-predominant irritable bowel syndrome, *IBS-D* diarrhea-predominant irritable bowel syndrome, *LCA* lithocholic acid, *SCFAs* short-chain fatty acids, *TNF-α* tumor necrosis factor-alpha, ↑ increased, ↓ decreased

D Neuroendocrine and Gut–Brain Axis Biomarkers

Beyond gut-localized disturbances, neuroendocrine mechanisms play a central role in irritable bowel syndrome (IBS) through dysfunction of the gut–brain axis. This network links the central nervous system, the enteric nervous system, and the hypothalamic-pituitary axis, and disruption of this network contributes to visceral hypersensitivity, a hallmark feature of DGBIs [35].

A key component of this system is neurotransmitter signaling, particularly serotonin (5-HT), which regulates gut motility, secretion and pain perception. Also, diminished GABA and altered GABAergic signals contribute to the pathogenesis of IBS-D [36]. In humans, studies showed that agents targeting the serotonin pathway interfere with the inflammatory cascade and cause amelioration in IBS symptoms [37]. Serotonin reuptake inhibitors, 5-HT3 antagonists, and 5-HT4 agonists have been used in the symptomatic management of IBS [35, 37]. 

The hypothalamic-pituitary-adrenal axis provides a critical link between psychological stress and gastrointestinal function. Psychological stressors, such as depression, anxiety, and major life traumas, are associated with increased IBS severity and contribute to altered gut physiology, including increased intestinal permeability and low-grade immune activation [35, 38]. Concurrently, stress-induced microbial shifts, characterized by reduced Lactobacilli and Bifidobacteria and an expansion of pathogenic organisms such as E. coli and C. jejuni, further disrupt epithelial integrity and gut homeostasis. The downstream effects are changes in epithelial function, mucus production and motility [38]. Functional MRI (fMRI) studies demonstrate that rectal distension in IBS patients is associated with increased blood oxygen level-dependent (BOLD) activity in brain regions involved in emotional and pain processing, that is, the limbic system, reflecting heightened central sensitivity in these patients [39].

Conversely, gut microbiota influence the gut–brain axis through metabolites such as SCFAs, which regulate immune responses, barrier function, and cortisol release [40]. Due to an increase in gut permeability in IBS, there has been a surge in interest in biomarkers such as Zonulin, which correlates with gut barrier dysfunction and symptom severity, supporting its potential role in patient stratification [41].

E Emerging and Digital Biomarkers

The emerging field of multi-omics can capture the intricate pathophysiology of IBS and pinpoint reliable biomarkers. The pursuit of overcoming current obstacles and developing effective biomarkers may benefit from innovative methods.

Each field of study provides a perspective on biological functionality: genomics identifies inherited susceptibility, transcriptomics reflects gene expression patterns that indicate active biological pathways, proteomics examines functional protein dynamics, and metabolomics further characterizes the composition and functional potential of the gut microbiota [42]. While each dataset is important, their combination can reveal the underlying mechanistic networks that contribute to symptom development and uncover cross-modal signatures that might be missed in single-layer evaluations.

Multi-omics revealed decreased *Bifidobacterium longum*, differences in SCFA, neurometabolites, and bacterial gene transcripts related to butyrate production and neuroendocrine hormones [43]. Temporal stability of the intestinal virome, associations between the virome and host colonic genes linked to immune responses and epithelial barrier function, associations of bacteriophage composition with clinical phenotype, as well as correlations with the primary bile acid chenodeoxycholate, have been investigated [44].

The incorporation of AI technology into these devices may also open new avenues for observing and customizing IBS treatments. In 2022, a study was published on this subject where the authors designed a smartphone application featuring AI for users to self-report on stool form assessments. The visual features utilized for the AI’s training included the Bristol Stool Scale, consistency, fragmentation, edge fuzziness, and volume. The results showed that the AI system was significantly more precise than the subjects’ own assessments when categorizing daily average Bristol Stool Scale scores as constipation, normal, or diarrhea (0.95 vs. 0.89) [45].

Looking ahead, AI-powered recommendation systems may enable personalized management of IBS to guide targeted omics and interventions. Multi-omics and AI represent a shift towards precision medicine in IBS, enabling accurate patient stratification and treatment.

### Methodological Challenges in IBS Biomarker Research

Although the Rome V criteria for IBS exhibit a moderate diagnostic capacity, a significant limitation in this field of study is the fact that no gold standard diagnostic test exists for IBS, against which a new diagnostic biomarker might be validated.

There is insufficient validation of the Rome criteria, and the majority of these validations relate the criteria to healthy individuals rather than organic GI disorders. One of the most important ways to improve the current paradigms of IBS diagnosis, assessment, and treatment is to find and validate actionable biomarkers [17].

The Rome criteria themselves have not been validated against a universally accepted diagnostic reference standard, and most validation studies have compared patients with IBS against healthy controls rather than individuals with organic gastrointestinal disorders. Moreover, because most available biomarker studies applied Rome III or Rome IV criteria, their diagnostic-performance estimates may not be directly transferable to the broader population identified using Rome V criteria. Prospective validation and recalibration of candidate biomarkers within Rome V-defined populations are therefore required [42, 46].

Beyond these methodological limitations, several practical barriers restrict the clinical applicability of current biomarkers. Many candidate biomarkers are influenced by factors unrelated to IBS itself, including diet, recent antibiotic exposure, medications such as nonsteroidal anti-inflammatory drugs, concurrent inflammatory or infectious conditions, and normal biological variability. These confounders may alter inflammatory, microbial, and metabolic marker concentrations independently of the underlying disorder, complicating their interpretation in individual patients. Importantly, few candidate biomarkers have demonstrated incremental clinical utility by meaningfully changing management, predicting treatment response, or improving patient-important outcomes beyond careful clinical assessment and limited guideline-directed testing. Consequently, nonspecific abnormalities may complicate rather than clarify clinical decision-making. False-positive results may prompt unnecessary investigations, increase healthcare costs, delay appropriate therapy, and heighten patient anxiety, whereas false-negative results may provide false reassurance and delay the recognition of coexisting organic disease. Biomarkers should therefore remain adjunctive to clinical assessment until their analytical validity, clinical utility, and impact on treatment outcomes have been prospectively established.

Biological tools that capture quantitative measurements of visceral pain and are valid across the spectrum from experimental animal models to humans are limited. The lack of rigorous surrogate endpoints for visceral pain has further hindered the translation of bench-to-bedside research in IBS, particularly in drug development, which continues to rely on endpoints defined by subjective patient-reported symptoms. Despite the body of knowledge and new treatment modalities that have accumulated over recent decades, IBS diagnosis and management continue to rely on symptom-centered strategies.

Although major society guidelines still support a positive symptom-based diagnosis with minimal testing, this approach is supplemented by empirical treatments chosen based on symptom phenotype and clinical history.

On the other hand, symptom phenotypes group people together who may have different and varied pathophysiologic processes. It follows that the high cost and burden of IBS, as well as the continued dissatisfaction of many patients and clinicians, are not surprising. Just 25% to 33% of respondents in a poll of more than 3000 IBS patients said they were “very satisfied” with using FDA-approved medications to treat their symptoms [47]. These observations show that although reliable clinical diagnoses can be achieved and a wide array of treatments now exist, there is a critical gap between establishing the diagnosis and delivering effective care [42]. The Indian consensus statements on IBS in adults also note the shortcomings of relying solely on a clinical classification system, while emphasizing the need for a multifaceted approach that includes better diagnostic criteria and validated biomarker use [48].

The British Society of Gastroenterology’s 2021 guidelines and a BMJ state-of-the-art review emphasize that while no single diagnostic test for IBS exists, we are entering an era of “actionable biomarkers” that may complement a symptom-based diagnosis [49]. Experts recognize that IBS treatment should move toward a mechanism-based approach in identifying the dominant pathophysiology in each patient and treating accordingly [49, 50]. Table 2 describes various methodological challenges in biomarker research in the context of IBS and strategies to address these challenges.

**Table 2:** Methodological challenges in IBS biomarker research

Methodological Challenges in IBS Biomarker Research	Strategies to address the challenge
No validated lab reference standards for IBS diagnosis	Using composite reference standards combining Rome criteria with exclusion of organic disease and long-term clinical follow-up
Biological heterogeneity and temporal variability in IBS pathophysiology	Using longitudinal sampling and multi-omics to define subgroups
Weak correlation between biomarkers and clinical endpoints	Studies can be linked to both patient-reported outcomes and markers of IBS, such as objective measures of visceral sensitivity or gut-brain function
Lack of standardized endpoints for biomarker-based trials	Developing consensus endpoints (gut permeability, immune activation, etc.) and corroborating them with regulatory agencies such as the FDA and EMA
Incomplete translation of animal or in vitro discoveries to human studies	Validating preclinical findings in human tissues, stool, and organoid systems
Predominance of cross-sectional study designs	Conducting longitudinal cohort studies to track biomarker trajectories across symptom fluctuations and treatment interventions
Insufficient integration of central, immune and microbial axes within biomarker frameworks	Using approaches such as gut-brain imaging, immune profiling, and microbiome studies to understand IBS pathophysiology

### Future Directions

Precision medicine is a term that was first introduced in 2011 as a method to develop targeted strategies to prevent, detect, and manage certain diseases. This encompasses a comprehensive system that accounts for neurohumoral factors, psychosocial elements, and patient outcomes.

Under ideal circumstances, a good biomarker must provide objective, precise, reproducible, and quantifiable measurements that correlate with patient outcomes and provide clinically meaningful information beyond what can be ascertained by patient history [17, 19]. Moving forward, biomarker work in IBS should prioritize endpoints that matter to patients and regulators, such as global symptom relief, pain relief, normalization of bowel habits, and overall improved quality of life.

Priority must be given to prospective and randomized trials, as opposed to cross-sectional case-control designs. Studies need to be modeled on panels that show a correlation between symptom severity and biomarker levels. To conduct research that can be translated to clinical practice, another effective strategy would be to embed standard pain and stool metrics alongside biomarker sampling to clarify whether a given marker is state dependent or simply epiphenomenal [42].

To move beyond symptom-based classifications, future studies should seek to define IBS endotypes anchored in gut–brain mechanisms (e.g. bile acid malabsorption, epithelial barrier dysfunction, low-grade mucosal immune activation, dysbiosis, visceral hypersensitivity, etc.). Multi-omics approaches combining genomic studies, metabolic trends, and microbiome patterns are particularly well-suited to generate such mechanism-based clusters that can be linked back to neurohumoral and psychological profiles [51].

From a regulatory perspective, biomarker development must involve clearly defined phases of validation and demonstration of clinical utility. There are several early-phase studies that need to specify patient subgroups and response trends in patient populations for the biomarkers to be integrated into routine use [52].

An actionable roadmap towards biomarker use would include: [52]

1. Consensus on mechanisms and corresponding candidate markers.
2. Standardized operating procedures for sample collection, processing, and analysis.
3. Multicenter prospective cohort studies.
4. Refinement of biomarker panels using machine learning models with reproducible pipelines.
5. Prospective evaluation of whether biomarker-guided management changes treatment selection and improves patient-centered outcomes relative to standard symptom based care.

**Fig. 1:**
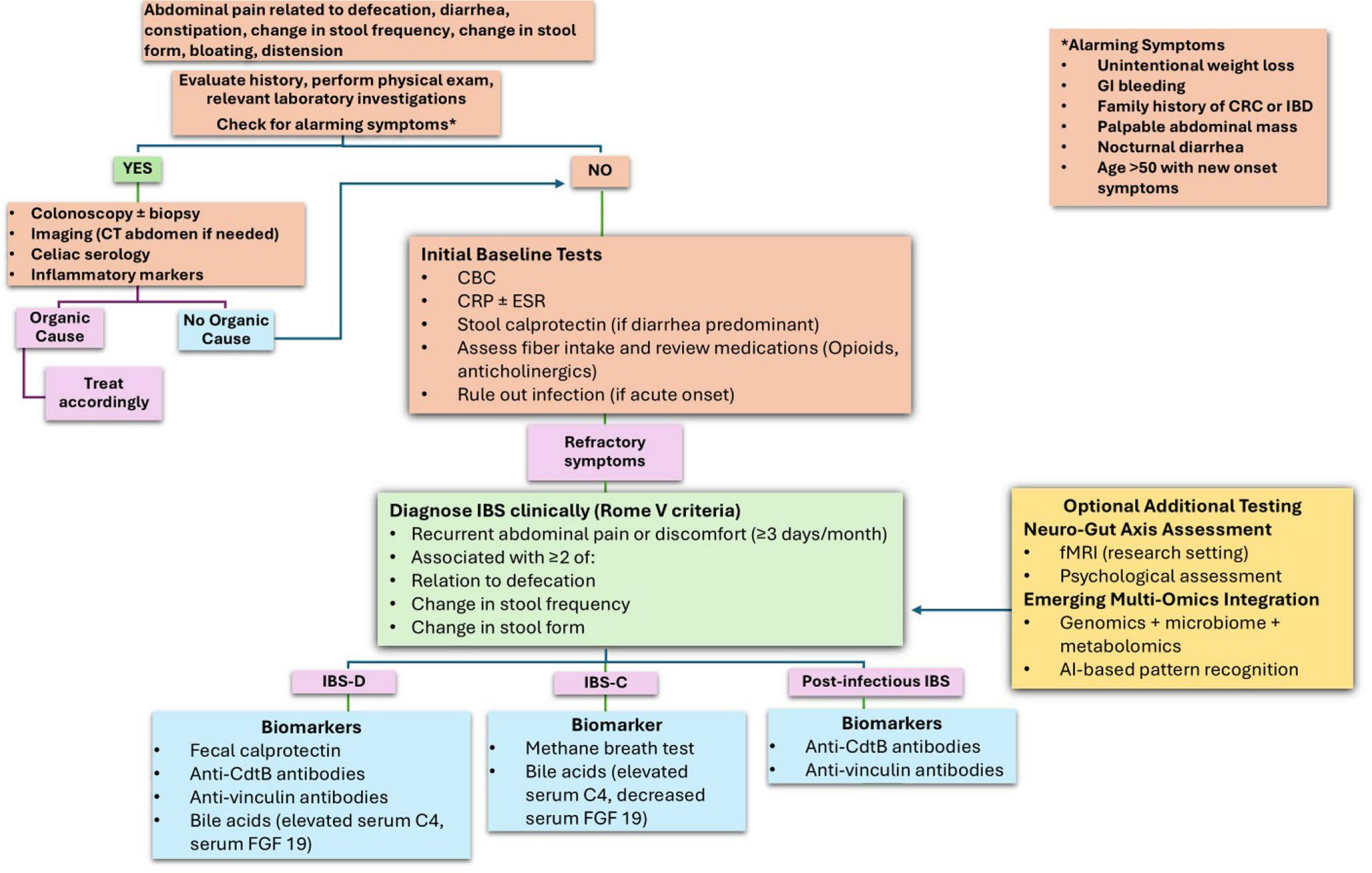
Integrating biomarker use into clinical workup for IBS. The figure presents a proposed clinical framework for IBS evaluation beginning with symptom assessment, and application of Rome V diagnostic criteria. It demonstrates the integration of inflammatory, microbial, metabolic, neuroendocrine, and emerging multi-omics biomarkers to support patient stratification and individualized management

We propose that clinically relevant biomarkers be incorporated into the diagnostic algorithm for irritable bowel syndrome, following initial clinical evaluation and exclusion of alarm features, to enhance diagnostic precision, reduce reliance on exclusion-based approaches, and facilitate a mechanism-driven individualized approach to management. We acknowledge that biomarkers must complement rather than replace existing clinical criteria. A workflow integrating biomarkers into subtyping IBS has been detailed in Fig. 1.

Ultimately, achieving an integrated clinical decision framework where symptom assessment can be validated using biomarker studies will improve treatment outcomes in IBS and position it to be a prototypical disorder that can provide a framework for several other medical conditions as well.

### Conclusion

Biomarkers in IBS are best utilized as adjuncts that enhance rather than replace a careful clinical diagnosis grounded in symptom-based criteria and exclusion of organic disease. An algorithmic approach using bowel habit patterns, alarm features, comorbidities, patient preferences, and biomarker panel utilization can refine risk stratification and guide therapy.

Most studies are limited by modest effect sizes, inter- and intra-individual variability, inconsistent assay methods, and the absence of universally accepted cut-off values or gold-standard reference tests, which constrains their immediate clinical utility. Streamlining the presently diverse sets of biomarkers will offset these restraints. Focusing on standardized research frameworks is an imminent need.

Looking ahead, the rapid evolution of emerging biomarkers spanning microbiome signatures to immune profiles signals a shift towards a mechanism-driven classification of IBS. As these markers are refined, they may offer a scalable and ultimately cost-effective alternative to extensive and cumbersome testing. The ability to noninvasively distinguish IBS subtypes would make diagnosing this disorder more efficient.

It is in this context that IBS, being a highly prevalent condition, can act as a model for developing and evaluating biomarkers in disorders of gut-brain interaction, leading to mechanistic insights in precision medicine and offering avenues for tangible improvements in patient care.

## Key References

- Soufan F, Ghosson A, Jaber R, Ghandour A, Uwishema O. The Gut-Brain Axis in Irritable Bowel Syndrome: Implementing the Role of Microbiota and Neuroimmune Interaction in Personalized Prevention-A Narrative Review. Health Sci Rep. 2025;8(4):e70660.
  - ○ This recent review synthesizes emerging evidence linking gut microbiota alterations, neuroimmune signalling, and gut–brain axis dysfunction in IBS. The authors highlight how these interconnected mechanisms may support personalized treatment strategies, reinforcing the growing importance of mechanism-based biomarkers in IBS.
- Shin A, Kashyap PC. Multi-omics for biomarker approaches in the diagnostic evaluation and management of abdominal pain and irritable bowel syndrome: what lies ahead. Gut Microbes. 2023;15(1):2195792.
  - ○ This article provides a comprehensive overview of multi-omics technologies and their potential to transform IBS diagnosis and management. The authors outline a pathway toward identifying biologically meaningful IBS endotypes and advancing precision medicine.
- Camilleri M, BouSaba J. Potential Value of Biomarker-Based Approaches for Evaluation and Management of Costly Functional Gastrointestinal Diseases. Clin Gastroenterol Hepatol. 2023;21(10):2462–72.
  - ○ This article reviews the challenges and opportunities associated with biomarker development in functional gastrointestinal disorders. It provides a practical perspective on validation, clinical implementation, and the steps required for biomarker-based approaches to become part of routine patient care.

## Supplementary Information

Below is the link to the electronic supplementary material.

Supplementary Material 1 (DOCX 30.0 KB)

Supplementary Material 2 (JPEG 73.4 KB)

Supplementary Material 3(JPEG 194 KB)

## Data Availability

No datasets were generated or analysed during the current study.
