# Supplementary material for: Biomarkers in Irritable Bowel Syndrome: Bridging Gut–Brain Mechanisms to Precision Care
Source: Curr Gastroenterol Rep. 2026 Aug 15;28(1):25. doi: 10.1007/s11894-026-01053-2 (PMC13477457; doi:10.1007/s11894-026-01053-2)
Supplement: Supplementary file 1 [file 11894_2026_1053_MOESM1_ESM.docx]

**Supplementary Tables**

**Supplementary Table 1: Categories of biomarkers and their proposed mechanisms**

| Biomarker Class | Examples | Proposed Mechanism |
| --- | --- | --- |
| Neuroendocrine markers | Serotonin, 5 HIAA, Substance P, cGRP | Intestinal motility, secretion and pain perception are affected by gut brain axis dysfunction (63) |
| Inflammatory markers | IL-6, IL-8, TNF-alpha, mast cell tryptase | Low grade immune activation leads to neuroimmune sensitization, abdominal pain and altered bowel habit (30) |
| HPA axis markers | CRF, ACTH, cortisol | Dysregulation of HPA axis leads to increased stress response, gut motility, increased intestinal permeability and visceral hypersensitivity (75,76) |
| Microbiome markers | SCFAs, reduced bifidobacteria | Dysbiosis affects fermentation, gas production bile acid metabolism and immune signalling (59) |
| Bile acid metabolism markers | Fecal bile acids, FGF-19 | Impaired bile acid signalling leads to IBS-C and excess bile acid leads to IBS-D (54) |
| Epithelial barrier and permeability marker | Zonulin, claudin-1, occludin | Luminal antigens activate neural and immune pathways due to increased intestinal permeability (78) |
| Metabolic markers | Ghrelin, Peptide YY, GLP-1 | Appetite, gut motility and visceral sensitivity are affected |
| Genetic and epigenetic markers | SERT polymorphism, cytokine variants, DNA methylation changes | Genetic susceptibility and epigenetic modification affects neurotransmission, stress response and immune activation |
| Central and peripheral neural markers | Functional MRI connectivity changes, autonomic tone markers  Nerve growth factor (NGF), TRPV1 | Central processing abnormalities maintain chronicity of symptoms.  Central and peripheral sensitizations of nociceptive pathways cause exaggerated response to normal gut stimuli 1(74) |

**Abbreviations:** 5-HIAA: 5-hydroxyindoleacetic acid; ACTH: adrenocorticotropic hormone; CRF: corticotropin-releasing factor; cGRP: calcitonin gene-related peptide; DNA: deoxyribonucleic acid; FGF-19: fibroblast growth factor 19; fMRI: functional magnetic resonance imaging; GLP-1: glucagon-like peptide-1; HPA axis: hypothalamic–pituitary–adrenal axis; IL: interleukin; NGF: nerve growth factor; SCFAs: short-chain fatty acids; SERT: serotonin transporter; TNF-α: tumor necrosis factor-alpha; TRPV1: transient receptor potential vanilloid 1

**Supplementary Figure Legends**

**Supplementary Figure 1: Conceptual framework of irritable bowel syndrome as a disorder of gut–brain interaction.** The figure illustrates major mechanisms implicated in IBS pathophysiology, including gastrointestinal dysmotility, visceral hypersensitivity, altered intestinal permeability, low-grade mucosal inflammation, and dysregulation of the gut microbiome–brain axis.

**Supplementary Figure 2: Pathways linking biomarkers to IBS pathophysiology.** The figure illustrates interconnected mechanisms involved in IBS pathogenesis, including neuroendocrine and gut–brain axis dysfunction, visceral hypersensitivity and altered gastrointestinal motility, bile acid and microbial metabolite dysregulation, microbial dysbiosis and post-infectious alterations, and epithelial barrier dysfunction with low-grade immune activation. Representative biomarker pathways associated with each mechanism are highlighted.
